# Reinventing Housing Care: Environmental Negotiations Made in Congregate Settings During COVID-19

**DOI:** 10.1093/geroni/igab046.1456

**Published:** 2021-12-17

**Authors:** Ian Johnson, Terri Lewinson

## Abstract

The COVID-19 pandemic prompted an urgent reconsideration of space and place within congregate housing. Research has only underscored the need for health-promoting physical alterations to residential environments (Peters & Halleran, 2020), but also generated lasting questions about the relationships between congregate environments and their residents, visitors, and workforce —among them, what ways can environments be negotiated to reduce risk (Dosa et al., 2020)? How can environments enact care for formal caregivers (Chen & Chavalier, 2021)? Who might be challenged by this care which may question the dangers associated with proximity (Lynn, 2020)? This symposium focuses on the ways stakeholders within congregate housing observed, related to, and negotiated changes to space and place during the pandemic. Paper 1 presents an organizational case study investigating provider perspectives of how housing and healthcare responses to COVID have shaped palliative care with unhoused patients during the pandemic. Paper 2 highlights the collaborative work of a multi-sector coalition working to address timely needs of residents in low-income senior buildings. Paper 3 reflects on the formation of a cross-national senior housing network and the interdisciplinary exchange of best practices and policy recommendations that emerged. The collective findings of these papers challenge previous notions of care in congregate environments, illuminate how provider networks respond to crises and share emergent knowledge, and consider how institutional decisions about the pandemic have re-placed and re-spaced provider and patient experiences. This symposium offers observations and strategies that may assist in envisioning successful congregate care during COVID-19 and beyond.

